# Activity Monitoring with a Wrist-Worn, Accelerometer-Based Device

**DOI:** 10.3390/mi9090450

**Published:** 2018-09-10

**Authors:** Wen-Yen Lin, Vijay Kumar Verma, Ming-Yih Lee, Chao-Sung Lai

**Affiliations:** 1 Department of Electrical Engineering, Center for Biomedical Engineering, Chang Gung University, Tao-Yuan 33302, Taiwan; d0421006@stmail.cgu.edu.tw (V.K.V.); cslai@mail.cgu.edu.tw (C.-S.L.); 2 Division of Cardiology, Department of Internal Medicine, Chang Gung Memorial Hospital, Tao-Yuan 33305, Taiwan; leemiy@mail.cgu.edu.tw; 3 Graduate Institute of Medical Mechatronics, Center for Biomedical Engineering, Chang Gung University, Tao-Yuan 33302, Taiwan; 4 Department of Nephrology, Chang Gung Memorial Hospital, Linkou, Tao-Yuan 33305, Taiwan; 5 Department of Materials Engineering, Ming Chi University of Technology, New Taipei 24301, Taiwan

**Keywords:** accelerometer, activity monitoring, regularity of activity, sleep time duration detection

## Abstract

This study condenses huge amount of raw data measured from a MEMS accelerometer-based, wrist-worn device on different levels of physical activities (PAs) for subjects wearing the device 24 h a day continuously. In this study, we have employed the device to build up assessment models for quantifying activities, to develop an algorithm for sleep duration detection and to assess the regularity of activity of daily living (ADL) quantitatively. A new parameter, the activity index (AI), has been proposed to represent the quantity of activities and can be used to categorize different PAs into 5 levels, namely, rest/sleep, sedentary, light, moderate, and vigorous activity states. Another new parameter, the regularity index (RI), was calculated to represent the degree of regularity for ADL. The methods proposed in this study have been used to monitor a subject’s daily PA status and to access sleep quality, along with the quantitative assessment of the regularity of activity of daily living (ADL) with the 24-h continuously recorded data over several months to develop activity-based evaluation models for different medical-care applications. This work provides simple models for activity monitoring based on the accelerometer-based, wrist-worn device without trying to identify the details of types of activity and that are suitable for further applications combined with cloud computing services.

## 1. Introduction

Acceleration due to the human activities is proven to be of great importance in epidemiological research and physical activity-based health assessment. MEMS accelerometer-based, wrist-worn devices, such as smart watches and smart wrist-bands, are becoming more and more popular for PA monitoring. These devices are not only being used to perform sleep assessment [1,2,3] but also for activity monitoring. Most of them provide number of steps and calorie consumption during the wearing period, and may even try to identify types of activities performed from the acceleration data measured by the accelerometer inside the devices. However, there are still unanswered questions such as: How accurate are these for the identified types of activities and calories burned, due to the fact the information is derived indirectly from acceleration information?

Having a good physically active life style can reflect one’s health condition and could be used to predict whether subjects might suffer from some diseases or not [4]. More importantly, having a regular life style and good sleep quality in the long run will even impact the subject’s health condition. However, there is no quantitative way to assess the regularity of daily life. Inadequate PA may result in many health problems and diseases related to the lungs, heart, etc. [5,6,7]. Tracking PA on daily basis provides valuable information related to the human body [8,9]. A number of platforms have been designed, implemented, and tested successfully in order to track subjects’ PA based on the wearable MEMS accelerometer [10,11]. Recent growth of IoT [12,13] and the capability of smart-phones in the past few years made PA recognition a dynamic field of study [14,15,16]. Bender et al. conducted an empirical study of various fitness devices such as Fitbit Flex, Fitbit Charge HR, Garmin Vívoactive, and Apple Watch to compare PA recognition accuracy and device performance [17].

Several previous works also proposed methods for activity identification and PA pattern analysis using external software, such as those executed on PCs or smart phones. For instance, Guillermo R. Oviedo et al. [18] have used GT3X ActiGraph accelerometer (Firmware 4.4.0, ActiGraph™, FortWalton Beach, FL, USA), and data were downloaded with the ActiLife 6 Software (v.6.12.0., ActiGraph™, Fort Walton Beach, FL, USA) for certain types of activity identification through activity patterns on PCs. Chelsea Dobbins and Reza Rawassizadeh [19] used principal component analysis feature selection (PCAFs) and correlation feature selection (CFs) on PCs to refine clustering of raw accelerometer data that had a positive effect on the computational burden that is associated with processing large sets of data, as energy efficiency and resource use is decreased, because less data is processed by the clustering algorithms, but a tremendous amount of raw data from devices is still required. A. K. Chowdhury et al. [20] have proposed the use of posterior-adapted, class-based weighted decision fusion to effectively combine data from multiple accelerometer-based devices for improving physical activity recognition. Nan Zeng et al. [21] have used NL-1000 pedometer and ActiGraph GT3X accelerometer for assessing the reliability of using motion sensors to measure children’s PA Levels in Exergaming. Matin Kheirkhahan et al. [22] have developed machine learning methods for identifying activity types and computing energy expenditures using standard statistical methods, as well as the bag-of-words (BoW) approach. However, this research poorly describes further applications for the results that have been analyzed.

In summary, these results are not found to be accurate enough or to have a close association with the timing information so that they could be considered for medical-care systems and clinical applications. Therefore, this research aims to bridge this gap and to develop simple mathematical models and algorithms for achieving parameters and results that are closely associated with actual on-set timing information. Moreover, the models and algorithms are simple enough to be easily deployed inside wrist-worn, accelerometer-based devices, and the results obtained are accurate and precise enough to be considered for medical-care systems and clinical applications.

Therefore, in this study we have proposed a methodology for the quantification of physical activities performed the daily life, i.e., activity index (AI), which is closely associated with human body acceleration [23]. These results can be used to categorize activities into 5 different levels, i.e., rest/sleep, sedentary, light, moderate, and vigorous activity states. Based on the AIs, a sleep duration detection algorithm has also been developed. Furthermore, by calculating the correlation coefficient for AIs on a daily or weekly basis, a quantitative method to express the regularity of daily life, i.e., the regularity index (RI), has been proposed in this study. By combining all these quantitative indices, such as activity, sleep duration, and regularity of ADL, this index can be used for many activity monitoring-based medical-care applications. Moreover, these results could be synced to smart phones for IoT applications with a greater capability of sensing human activity ubiquitously and unobtrusively through advancements in miniaturization and sensing abilities. This could unleash a systematic methodology in mobile-health for the prevention and early detection of chronic diseases.

## 2. Materials and Methods

**Figure 1:**
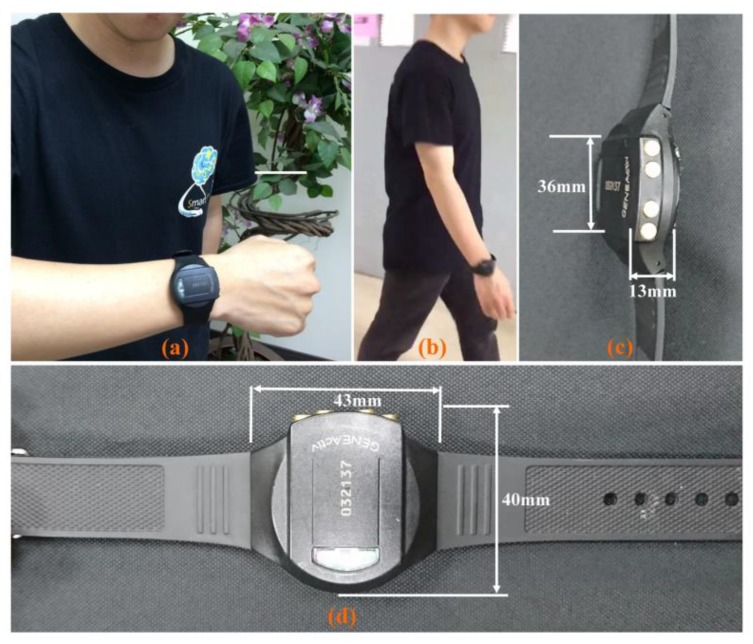
(**a**) Accelerometer-based GENEActiv device, Activinsights Ltd., Huntingdon, UK. (**b**) Walking subject with GENEActiv device on right-wrist. (**c**) Physical dimensions and side view of the GENEActiv device. (**d**) Top view of the device.

This study was reviewed and approved by the institutional review board (IRB) of the Chang Gung Memorial Hospital, Taiwan, R.O.C. An accelerometer-based, wrist-worn device (GeneActiv, Activinsights Ltd., Huntingdon, UK, as in Figure 1) has been used to record wrist movement acceleration in three orthogonal directions along with timestamp and body temperature. This device is equipped with MEMS-based accelerometer, which was set for a sensing range of ±8 g at a 12-bit digital resolution (i.e., 3.9 mg resolution, 1 mg = 1/1000 g), in which ‘g’ is the gravitational force. Even though the sampling frequency of this device was configurable, the sampling frequency was set to 20.0 Hz, as it was used to monitor ADL and also to prevent too much data were generated due to high sampling rate. Ten normal subjects were chosen and have been asked to wear the device for 24 h a day during their normal routine. After a maximum of 30-days, data were downloaded at the data-server station, and the subjects were given another device immediately for continuous data recording. 

Downloaded data were then further analyzed and interpreted. A mathematical model has been proposed and implemented for the data processing and parameters for quantifying and assessing PAs that were defined, quantified, and evaluated. A brief description about data analytical models are as given below.

### 2.1. Data Analytical Models

With the fact that the kinematic energy associated with human activities can be given as (1),

(1) $$E=\frac{1}{2}mv^{2}$$

in which ‘m’ is the human’s body mass moving with a velocity of ‘v’. Since $v=a\cdot \Delta t+v_{0}$, in which ‘*a*’ is the moving acceleration of the body in the time interval of ‘Δt’, and ‘$v_{0}$’ is the initial velocity. If the movement starts from rest position, then the initial velocity, ‘$v_{0}$’, can be taken as 0, and v=a·Δt. Now, the kinematic energy in (1) will be proportional to the square of acceleration, as in (2), assuming that the subject’s mass is constant over the period Δt.

(2) $$E\propto \;a^{2}$$

The magnitude of acceleration, $A_{j}$, at the data instance *j* could be calculated as in (3), and since only the magnitude is calculated, the impact of the orientation of accelerometer will be eliminated.

(3) $$A_{j}=\sqrt{\left(a_{x{\_}_{j}}^{2}+a_{y{\_}_{j}}^{2}+a_{z\_j}^{2}\right)}$$

In (3), $a_{x\_j}$, $a_{y\_j}$, and $a_{z\_j}$ are the raw data from accelerometer along the X-, Y-, and Z-axis sampled at the time instance *j*. The standard deviation, σ, of the acceleration magnitude within a pre-defined epoch period can be calculated as in (4).

(4) $$\sigma =\sqrt{\frac{1}{N}\sum_{j=1}^{N}{\left(A_{j}-\mu \right)}^{2}},\;\mathrm{where}\;\mu =\frac{1}{N}\;\left(A_{1}+\;A_{2}+\;A_{3}+\cdots \cdots \cdots +\;A_{N}\right)$$

In (4), *N* is total number of acceleration data points measured in the epoch period, and μ is the mean value of the total acceleration within that period. Since the acceleration measured by the accelerometer also contains gravity force all the time; therefore, the average of total acceleration within a short period can be considered as the gravity. Hence, deducting the acceleration’s mean value ‘μ’ from the magnitude of acceleration $A_{j}$ measured by accelerometer will give the net acceleration of the body movement. Therefore, the square of standard deviation, σ, calculated in (4) can be considered as the average of the square of total net acceleration within the epoch period, such that the human kinematic energy will also be proportional to the square of standard deviation, σ, as in (5).

(5) $$E\;\propto \;a^{2}\;\propto \;\sigma^{2}$$

Since the square of standard deviation, $\sigma^{2}$, over the epoch period is very small and usually less than 1, so the standard deviation, σ, is taken into consideration in the model proposed in this study for the larger value represented. Regarding the results, an activity index (AI) is proposed as the summation of the standard deviation, σ, over a desired time interval, as shown in (6).

(6) $$AI=\sum_{k=1}^{M}\sigma_{k}$$

in which *M* is the total number of epoch periods within the time interval and $\sigma_{k}$ represents the standard deviation of acceleration in the *k*-th epoch period within that time interval. In the implementation of the data analytical models described here, a 5-s epoch period is considered. The choice of 5-s epoch period typically will not cover more than two activities within that epoch period and will still generate manageable amounts of data in the computation. Since the sampling frequency of the accelerometer in the device was set to 20 Hz, within the 5-s epoch period, there will be 100 measured data points, i.e., *N* = 100 as in (4). To generate a minutely-wise AIs, i.e., choosing one minute as a basic time interval, then there will be 12 epoch periods in the time interval, and hence *M* = 12 as in (6).

### 2.2. Categorization for Different Levels of Activities 

**Figure 2:**
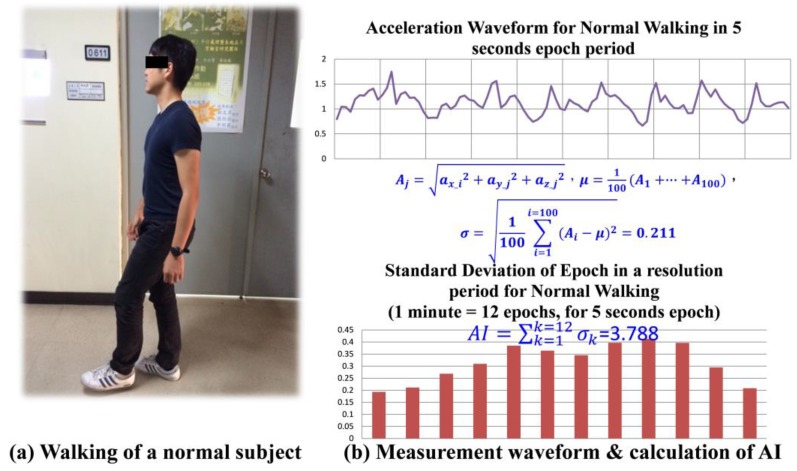
(**a**) Photo of walking test and (**b**) acceleration waveform of measured activity and calculation of AI.

With the AI introduced in Section 2.1, testing of 14 different activities of daily life (ADL) and each activity lasted for 3 min was conducted with 10 normal subjects wearing the device. These activities included sleeping, sitting and watching TV, sitting and reading newspaper, sitting and web browsing, housekeeping, driving, walking—no hand-swing, walking—with hand-swing, upstairs—no hand-swing, upstairs—with hand-swing, downstairs—no hand-swing, downstairs—with hand-swing, jogging—no hand-swing, and jogging—with hand-swing. Among these 10 different normal subjects under test, only the types of activities to perform for testing were instructed; no detailed constraints, such as numbers of body turns during the sleep, moving (swing) frequency of the arms, walking speed, etc., were asked to be followed. Figure 2a shows a normal subject walking with hand-swing during the test, and Figure 2b depicts the magnitude of acceleration recorded over an epoch period, 5 s, for the activity, and a sample calculation for the standard deviation of acceleration recorded within that period is shown as Figure 2b. Figure 2b shows that the 12 standard deviations, σ′s, calculated in a one minute time interval, and by accumulating such 12 σ′s, a minute-wise activity index, AI, for the walking with hand-swing is obtained.

Among these 14 ADL tested, sleeping is considered as a typical rest/sleep level of activity; the activities sitting and watching TV, sitting and reading newspaper, and sitting and web browsing, which were all performed in sitting position, are sedentary level activities; housekeeping, driving, and walking without hand-swing are classified as light level of activities; walking with hand-swing and up and down stairs, with or without hand-swing, are all moderate levels of activities; finally, jogging with or without hand-swing are considered vigorous levels of activity.

### 2.3. Sleep Duration Detection

**Figure 3:**
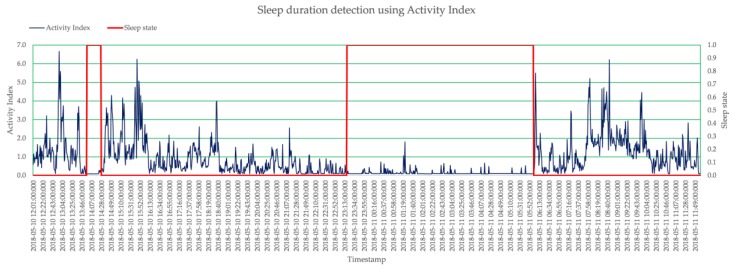
24 h minute AI pattern with sleep state identified.

Figure 3 shows the 24-h AI pattern of a subject. These AIs were calculated minute-wise, and hence there are 1440 AI values within 24-h period of daily life. The 24-h period started from 12:00:00.000 on the day and end at 11:59:59.950 on the next day, so that the typical sleep duration during the night can be covered in a 24-h minute-wise AI pattern completely. From this pattern, it is very clear that around 14:07, the level of AI is very low, as the subject was taking a nap, and the duration between 23:13:00 to 05:52:00 of the next day is also low, as the subject was sleeping.

Although detecting sleep duration with accelerometer has been practiced for a long time, neither methods use the proposed AI for recognition nor results are precise and accurate enough so that they can be used for clinical applications. Sleep duration detection is of utmost importance in the diagnosis of diseases like insomnia, drowsiness, and other sleep-related disorders. A smart sleep duration detection algorithm has been developed and is introduced in more detail below. The algorithm is based on the AI models that have been proposed in previous section.

**Figure 4:**
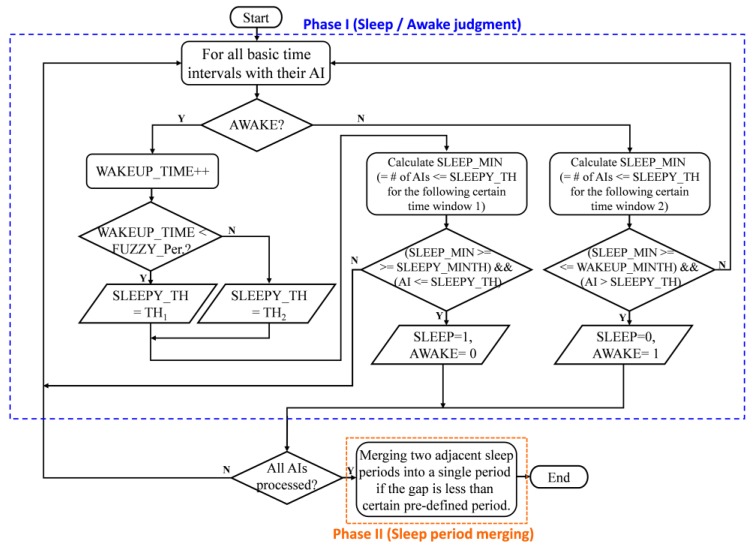
Flow chart of the proposed smart sleep duration detection algorithm based on AI model.

The flow chart of the sleep duration detection algorithm is shown in Figure 4. The proposed algorithm consists of two phases. Phase I is to judge whether the subject in current time interval is in sleeping or awake state. In this phase, the proposed algorithm will thoroughly process all of the AIs corresponding to all basic time intervals. If the previous time interval is in awake state, the algorithm will judge if the subject falls asleep in the current time interval; otherwise, it will see if the subject wakes up during this time interval. 

A subject is said to be in a fuzzy period when the subject just woke up within certain period of time within the sleep duration, and there are two different criteria to judge if the subject falls asleep in current time interval or not based on if the subject is in a fuzzy period. This reflects the fact that a subject will be most likely to fall asleep again when the awaking duration is not long enough. As a result, relaxed criteria (higher threshold value considered for current AI) could be used to judge if the subject falls into asleep or not when the subject is in a fuzzy state. Otherwise, stricter criteria (lower threshold value could be considered for current AI) is used to detect if the subject falls asleep. Besides the threshold value used, the criteria for a subject to fall asleep in current time interval requires the number of AIs, i.e., SLEEP_MIN, that is lower than the threshold value, SLEEP_TH, within the following certain time window 1 to be more than some pre-defined value, SLEEP_MINTH, and also that the AI of current time interval be lower than the threshold value. 

Similarly, judging if the subject wakes up in current time interval requires meeting wake-up criteria. It is defined as the number of AIs, i.e., SLEEP_MIN, that are lower than the threshold value within the following certain time window 2 to be less than some pre-defined value, WAKEUP_MINTH, and for the AI of current time interval to be greater than the threshold value.

After all the AIs of the all basic time intervals have been processed thoroughly, all the sleeping periods are detected. Then, the algorithm goes into Phase II. Phase II is to merge any two adjacent sleeping periods into a single period if the duration is less than certain pre-defined time. This phase could eliminate the results of fragmented sleep durations detected in Phase I that falsely indicate bad sleep quality. Typically, people will consider they sleep for a whole period of time but often wake up during sleep instead of having fragmented sleep durations. Phase II considers this situation and, as a result, will have more matched and accurate sleep duration with the subject’s intuitive cognition.

As per the assessment of AI under rest/sleep level of activities in previous section, the AI is less than 0.1 within the 1-min time interval under rest/sleep status. Hence, AI value of 0.1 is used in the very beginning of the sleep duration detection algorithm to judge if that time interval is in AWAKE state or not.

### 2.4. Quantification of Regularity of ADL

**Figure 5:**
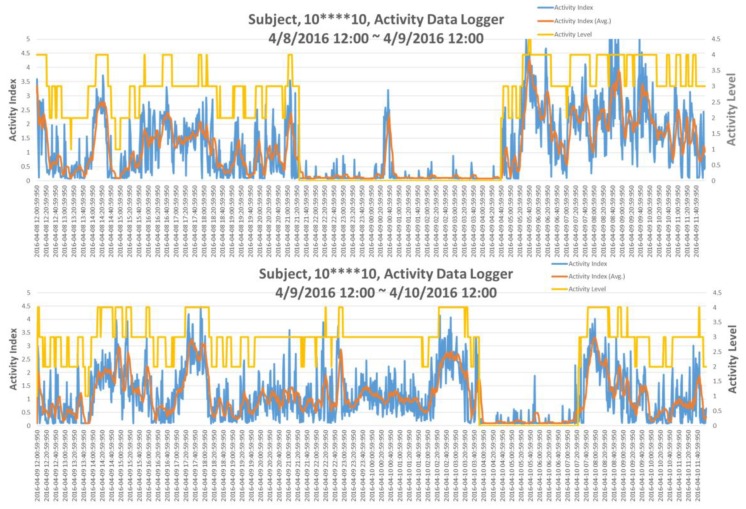
The AI pattern plots of a single subject over two consecutive days.

In the IRB testing, a typical situation was that a single subject with un-regular living patterns in two consecutive days has been observed; this is shown in Figure 5. Indeed, the subject went to the emergency room (ER) for urgent health situation on the next day. Apparently, as in top of Figure 5, the subject’s sleep duration was between 21:21:01 on the day to 04:41:01 of the next day. However, according to the AI pattern of the next day, as in the bottom of Figure 5, the subject went to bed around 04:00:01 in the morning and woke up at 07:21:01 with only very short sleep duration and also in extremely irregular time slot. This situation suggested that the subject’s life style on these two consecutive days was extremely irregular, which might result in his visiting ER the next day. This observation motivated the need for a quantification assessment of regularity of ADL. 

**Figure 6:**
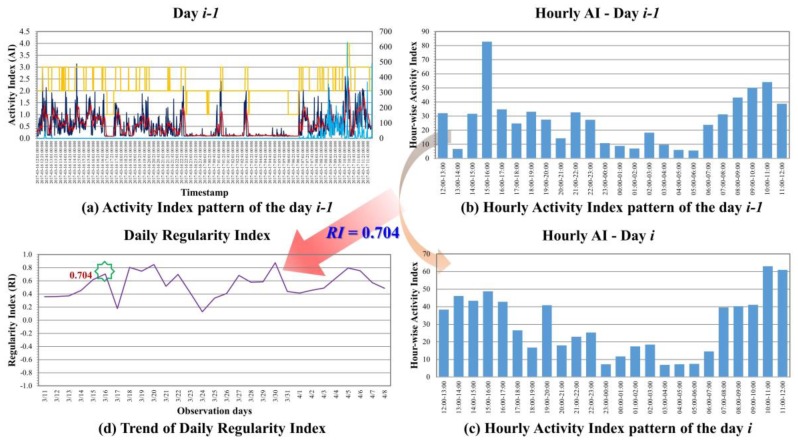
(**a**) Minute-wise AI pattern of day *i−1*; (**b**) hourly AI pattern of day *i−1*; (**c**) hourly AI pattern of day *i;* and (**d**): RI trend.

As shown in Figure 6a, there are 1440 data points of minute-wise AIs in a 24-h day, which raises significant complexities for further processing. Therefore, hour-based AI patterns were generated by taking the cumulative values of 60 min-wise AIs patterns into the total AI within an hour. As a result, hourly AI patterns of the day are generated, as shown in Figure 6b. By finding the correlation coefficient between the hourly AI patterns of day *i−1* and day *i*, as shown in Figure 6c, the result can indicate the regularity of ADL of the day *i* with respect to the previous day *i−1*, namely, day-to-day Regularity Index (RI). Similarly, if the hourly AI patterns of day ***i*** are compared with the patterns of the day one week before, i.e., day *i−*7, then it is called week-to-week Regularity Index. Since the range of the correlation coefficient is in between −1 and +1, the result of +1 means the ADL pattern of day *i* is the same as day *i−1*. 0 stands for totally uncorrelated and −1 stands for totally inversely correlated. With the continuous monitoring of days for human’s ADL, the trend for the regularity of that subject’s living style can be plotted as in Figure 6d.

## 3. Results

As described in Section 2.2, 14 different types of daily physical activities (PAs) were performed by 10 normal subjects with GeneActiv devices worn on their wrists. Maximum and minimum values of AI for a specific PA have been identified; furthermore, the mean value of AI for that type of PA was calculated. As there were no detailed constraints, such as numbers of body turns during sleep, moving (swing) frequency of the arms, walking speed, etc., for any specific type of activity that were followed in the test, the range of maximum and minimum AIs for some types of activities were quite large. However, they can still be categorized into different levels of activity based upon their mean AI values; furthermore, the AI value falls between the specific maximum and minimum ranges, as shown in Table 1. For instance, mean AI value less than 0.1 indicates that the subject under observation was sleeping or resting, similarly, mean AI value between 0.1 and 0.5 will be considered as subject was performing sedentary types of PAs. For light PAs, the mean AI falls between 0.5 and 2.0. Moderate PAs could be considered as having mean AI in the range of 2.0~4.0. Additionally, all PAs having mean AI greater than 4.0 could be considered as performing the vigorous activities. The level of activities that have been categorized are shown as the yellow line in Figure 5, with rest/sleep leveled at 0 and vigorous activities leveled at 4 respectively.

With the same acceleration data, a 24-h minute-wise AI pattern starting from the noon of a day to the noon of next day were analyzed by the proposed smart sleep duration detection algorithm to detect the sleep duration with a precision up to 1 min. The red line in Figure 3 shows the identified sleep states, and it represents ‘awake’ state by ‘0’ and ‘sleep’ state by ‘1’. As stated, through the phase II—sleep period merging, no more fragmented sleep periods were generated due to the bad sleep quality. The sleep quality can also be quantified by calculating the average minute-wised AI during the sleep duration. Larger value means there were more activities during the sleep, and hence it stands for bad sleep quality. Smaller values mean few activities occurred during the sleep, which indicates good sleep quality.

Regularity Index (RI) of ADL, which is the correlation coefficient between the hourly AI patterns of day *i−1* and day *i*, effectively represents the regularity of PAs on hourly-basis on the day *i* and is in the range of ±1. Table 2 lists an example of one-week data for a subject, arranged from noon to noon of next day; hourly AIs are listed in different columns with respect to the dates. RI value of +1 means the ADL pattern of the day *i* is the same as of day *i−1*; 0 stands for totally uncorrelated, and −1 stands for inversely correlated.

## 4. Discussions and Conclusions

With the proposed analytical model representing the quantified PAs and different levels of PAs identified in this study, several parameters can be used to evaluate the status of subject with the activity-based information. As shown in Table 3, besides the AI/min. (minute-wised activity index) and day-to-day RI introduced previously, a simple total summation of the AIs over the whole day, i.e., T_AI, can simply indicate the total amount of activities within the day performed by the subject. The SL_T and SL_Q representing the sleep hours and sleep quality can indicate the amount of time that the subject was asleep or in rest during the day and the quality of the sleep/rest, respectively. T_[level of activity] stands for the total number of hours that a subject can perform certain level of activities. For example, if the information of the duration that a subject performed activities above certain level (including the specific level) is required, then it can be calculated with T_[Level] + T_[Level+1] + … + T_[4]. The last parameters, A[x]_T_[level of activity], which are of interest, constitute the time duration that a subject performed certain level of activities within certain time window from when the subject was awoke. This may indicate the capability of that subject performing certain level activities within the time window from getting enough rest, i.e., period from when they awoke. For example, in some clinical applications, the capability (evaluated by the time duration) that people can perform activity levels greater than or equal to 3 (moderate level) within 3 h when they awake may be a strong indication representing their lung condition.

With these parameters or even by combining these, they could be strong indicators for many medical-care applications by analyzing either the changes of the long-term trends of these parameter or some machine learning algorithms. In this study, analytical models and methods were proposed to perform activity-based monitoring with accelerometer-based wearable devices. A new parameter, activity index (AI), has been introduced and used to categorize different PAs into 5 levels. Another new parameter, regularity index (RI), has been proposed to represent the degree of regularity of ADL. It provides a quantitative measurement of the regularity of living and can be used for many quantified risk assessments of certain diseases. The proposed models and calculations are simple enough to have them implemented into existing accelerometer-based wearable devices. Hence, they are extremely suitable for further applications combining cloud computing services and IoT-based online health monitoring platform, or for monitoring the health condition of a patient discharged from the hospital and predicting their next re-hospitalization by observing varying patterns in ADL.

## Figures and Tables

**Table 1:** Classification of different PAs based upon AI.

Daily Life Activities	Level of Activities	Maximum	Minimum	Mean
**Rest/Sleeping**	**REST/SLEEP**	0.096975	0.082482	0.088523
**Sit-Watching TV**	**SEDENTARY**	0.345286	0.092849	0.186028
**Sit-Reading News paper**	**SEDENTARY**	0.466089	0.090514	0.301787
**Sit-Web browsing**	**SEDENTARY**	0.12198	0.100102	0.111355
**Housekeeping**	**LIGHT**	1.604074	0.829076	1.222838
**Driving**	**LIGHT**	1.378413	0.976756	1.171664
**Walking-no Hand-Swing**	**LIGHT**	2.334001	0.648251	1.59335
**Walking-w/Hand-Swing**	**MODERATE**	3.973975	2.048219	2.541614
**DownStairs-no Hand-Swing**	**MODERATE**	8.124398	1.673799	3.890505
**DownStairs-w/Hand-Swing**	**MODERATE**	3.40835	1.243286	2.415157
**UpStairs-no Hand-Swing**	**MODERATE**	2.602795	2.352814	2.515802
**UpStairs-w/Hand-Swing**	**MODERATE**	2.464628	2.308799	2.393241
**Jogging-no Hand-Swing**	**VIGOROUS**	12.95352	4.437786	8.742815
**Jogging-w/Hand-Swing**	**VIGOROUS**	7.27138	4.832407	6.033422

**Table 2:** A sample of one-week data, illustrating assessment of RI.

Hour\Date	9 May 2018	10 May 2018	11 May 2018	12 May 2018	13 May 2018	14 May 2018	15 May 2018
**12:00:00**	91.6985	7.9372	52.1803	39.1756	16.1481	120.7643	7.4088
**13:00:00**	68.0396	155.7581	104.2664	20.2958	190.7752	68.5014	59.4915
**14:00:00**	60.9835	132.4570	209.2683	47.8527	165.0358	24.0670	79.0501
**15:00:00**	127.9568	97.1954	152.4025	63.6552	72.7410	17.4707	110.0526
**16:00:00**	50.0016	37.5953	5.2637	20.1774	34.5341	125.6139	55.4181
**17:00:00**	52.6829	63.8394	5.0618	40.1613	60.5130	50.6716	35.2950
**18:00:00**	58.4110	44.1087	15.8595	39.5041	35.8857	16.3036	43.0587
**19:00:00**	30.3024	11.9919	23.8395	20.0936	34.3655	24.9894	19.2359
**20:00:00**	32.9313	44.0399	19.5678	19.0029	49.1668	21.5088	42.3071
**21:00:00**	25.9622	20.2555	32.2234	16.1118	45.0717	18.5637	26.7250
**22:00:00**	13.7704	16.6188	12.7696	9.2744	26.0573	9.6644	16.1690
**23:00:00**	8.8460	11.4903	13.3283	9.6019	16.6092	8.3738	10.8001
**00:00:00**	9.1765	8.0131	11.8799	9.2327	12.3928	7.2355	7.0924
**01:00:00**	11.7781	13.9831	9.7203	7.5271	18.6474	12.4477	7.9296
**02:00:00**	7.1743	25.7008	6.9460	10.2525	9.9166	7.7463	15.6859
**03:00:00**	6.9395	16.4114	8.0269	7.3836	13.6776	7.3144	8.6616
**04:00:00**	6.9992	18.5755	10.9319	6.9433	13.1182	7.3127	11.0475
**05:00:00**	6.6685	66.1852	33.5049	10.2133	87.0340	81.8432	69.0056
**06:00:00**	51.9434	60.5493	53.4563	45.3398	28.0925	201.7091	43.4253
**07:00:00**	60.3498	146.4305	152.9999	199.2066	72.4182	161.1731	138.3872
**08:00:00**	146.0198	185.8036	184.6546	132.9641	95.0854	98.3405	239.4251
**09:00:00**	109.5714	126.9927	105.3715	178.1697	16.5196	96.5270	150.3620
**10:00:00**	50.1309	97.9686	48.7204	84.0562	42.8646	131.4003	77.6424
**11:00:00**	53.7380	95.5183	34.1327	74.0714	37.0004	53.0020	78.3121
**RI**	**-**	**0.7078**	**0.8386**	**0.6484**	**0.1430**	**0.1105**	**0.4395**

**Table 3:** Parameters used for activity-based monitoring.

Parameters	Meanings
AI/min.	Activity Index/min.—minute-wised activity index
T_AI	Total Activity Index of a Day—summation of all the minute-wised AIs within a day
D_RI	D-to-D Regularity Index—regularity index between the day and the day before
SL_T	Sleep hours—number of hours in sleeping/resting
SL_Q	Sleep quality—average minute-wised AI in sleep duration
T_[Level of Activity]	Hours of Activity [Level]—total time duration performing certain level of activities in a day
A[x]_T_[Level of Activity]	Duration of Activity [Level] within [x] hours after awake—time duration of performing certain level of activities within [x] hours after awake
